# A Qualitative Study on Caregiving Burden & Sleep Pattern: Coping Among Long-Distance South Asian Caregivers

**DOI:** 10.1093/geroni/igaf122.3314

**Published:** 2025-12-31

**Authors:** Dinesh Karki, Srujana Chekuri, Akankshya Chataut, Julie Blaskewicz Boron

**Affiliations:** University of Nebraska Omaha, Omaha, Nebraska, United States; University of Nebraska Omaha, Elkhorn, Nebraska, United States; University of Nebraska Omaha, Omaha, Nebraska, United States; University of Nebraska Omaha, Omaha, Nebraska, United States

## Abstract

Long-distance caregiving (LDC) is a unique experience which mitigates some caregiving disturbances (e.g., nocturnal wandering), but exacerbates others (e.g., care recipient monitoring). LDC may increase stress and disrupt regular sleep, negatively affecting the caregiver’s health and quality of life. Sleep disturbances in LDC result from a complex interplay of environmental conditions, personal responsibilities, and coping strategies. This study explored how external factors—including neighborhood, community, and social support—affect sleep among South Asian (SA) long-distance caregivers responsible for companionship, financial assistance, and emotional support. SA Caregivers (N = 78) aged 19-73 (M = 26; SD = 7). were recruited for a larger needs assessment. A subset of participants (n = 21, MAge=26, Male=80.1%) completed one of five focus group sessions via Zoom. Thematic analysis was conducted independently by two reviewers using MAXQDA for inductive coding. Results revealed that caregivers employed diverse coping strategies (e.g., white noise machines, earplugs, adjusted sleep schedules) to mitigate sleep disruptions, however, these only partially addressed long-term concerns. Most (71.4%) reported that strong family and community support systems were critical for reducing caregiving stress and improving sleep quality; neighborhood support was also identified as important by 23.8% of caregivers. In contrast, 19% of caregivers with limited social support reported higher stress and poorer sleep outcomes. These findings highlight the importance of interpersonal networks for fostering resilience and well-being for long-distance caregivers. Addressing contextual factors, implementing effective coping strategies, and strengthening community engagement are essential for enhancing long-distance caregivers’ sleep quality and overall health. Future research should examine these relationships in other cultural identity groups.

